# Radiation Crosslinked Smart Peptide Nanoparticles: A New Platform for Tumor Imaging

**DOI:** 10.3390/nano11030714

**Published:** 2021-03-12

**Authors:** Atsushi Kimura, Miho Ueno, Tadashi Arai, Kotaro Oyama, Mitsumasa Taguchi

**Affiliations:** 1 Takasaki Advanced Radiation Research Institute (TARRI), National Institutes for Quantum and Radiological Science and Technology (QST), 1233 Watanuki-Machi, Takasaki, Gunma 370-1292, Japan; kimura.atsushi@qst.go.jp (A.K.); oyama.kotaro@qst.go.jp (K.O.); 2 Graduate School of Science and Technology, Gunma University, 1-5-1 Tenjintyo, Kiryu, Gunma 376-8515, Japan; t201a020@gunma-u.ac.jp (M.U.); t170a006@gunma-u.ac.jp (T.A.)

**Keywords:** peptide, nanoparticle, radiation crosslinking, γ-rays, tumor diagnostics

## Abstract

Nanoparticles have been employed to develop nanosensors and drug carriers that accumulate in tumors. Thus, it is necessary to control the particle size, surface potential, and biodegradability of these nanoparticles for effective tumor accumulation and safe medical application. In this study, to form a nanoparticle platform suitable for diagnostic and drug delivery system (DDS) applications, peptides composed of aromatic amino acid residues were designed and synthesized based on the radiation crosslinking mechanism of proteins. The peptide nanoparticles, which were produced by γ-ray irradiation, displayed a positive surface potential, maintained biodegradability, and were stable in water and phosphoric buffer solution during actual diagnosis. The surface potential of the peptide nanoparticles could be changed to negative by using a fluorescent labeling reagent, so that the fluorescent-labeled peptide nanoparticles were uptaken by HeLa cells. The radiation-crosslinked nanoparticles can be applied as a platform for tumor-targeting diagnostics and DDS therapy.

## 1. Introduction

There are various diagnostic methods for tumors in medical treatment; however, some of them impose drug side-effects on the patients. Drug delivery systems (DDS) that efficiently deliver drugs to tumors have made great development in solving these problems. Moreover, hard and soft functional nanoparticles that easily accumulate in a tumor (passive targeting) have been developed in recent years [1,2,3,4,5]. Nanoparticle-based contrast agents that can be applied to common biomedical imaging devices such as fluorescence imaging, magnetic resonance imaging (MRI), computerized tomography (CT), positron emission tomography (PET), and single-photon emission computerized tomography (SPECT) have also been developed [6]. Additionally, peptide-based magnetic nanoparticles with targeting and drug release properties through external magnetic fields have been reported [7,8]. Many functionalized nanoparticles respond to the internal environment, such as pH, redox, temperature, and enzymes [9,10]. The detailed structure of nanoparticles has also been investigated by small-angle X-ray scattering and cryogenic transmission electron microscopy [11]. Accumulation of these nanoparticles to human tumors has been studied, and their retention time and distribution in the tumor depend on the tumor type and stage. Thus, the size of these nanoparticles is adjusted to target the tumor cells [2,12] and to allow them to pass through the perforated blood vessel of the tumor [10,13]. These particles therefore attach to the tumor cells and are subsequently taken up into the cells mainly via endocytosis. Cabral has also reported that smaller size nanoparticles are more preferentially taken up by refractory pancreatic tumor cells [14].

Nanoparticle cell uptake also depends on the surface potential, which is measured as a Zeta potential by the electrophoretic light scattering (ELS) method. Positively charged nanoparticles induce strong interactions with the anionic cell membrane surface and are immediately taken up by the cell by electrostatic adhesion-mediated targeting [5,14,15]. On the other hand, neutral and negatively charged nanoparticles can be transferred across longer distances in the tumor tissue than the positively charged nanoparticles. Thus, it was reported that positive chitosan-based nanoparticles, ~200 nm in size, were taken up by the cell, escaped from the lysosomes, and then localized around the cell nucleus [16]. On the other hand, neutral and negative chitosan particles tend to localize in the cell lysosomes. The cell uptake of cerium oxide-based nanoparticles, which coat polymers with positive, neutral, and negative charges, have been investigated. The study revealed that the positive and neutral nanoparticles were taken in the normal cell line, while the negative nanoparticles were taken up by the tumor cell line [17]. On the other hand, 150 nm-sized nanoparticles with a trace negative charge tended to effectively accumulate in the tumor [18]. These reported results indicate that the nanoparticle surface potential significantly affects their accumulation and stability in the cell.

Apart from the particle size and surface potential, the amino acid composition in nanoparticles is also an important factor for cell adhesion and accumulation to the tumor cell. The targeting rate of 300 nm nanoparticles toward human umbilical vein endothelial cells (HUVEC) was enhanced by peptide functionalization modification, with the cell adhesion amino acid sequence RGD, of the particle surface [19]. The elimination of nanoparticles from the body is a significant factor, and their rapid expulsion from the body is encouraged to reduce side effects. However, most nanoparticles applied as DDSs for in vivo diagnosis are >7 nm in size, and thus their renal extraction via the glomerulus is difficult [15,20]. Moreover, it is difficult for these particles to accumulate in the liver for subsequent decomposition or excretion via the hepatobiliary tract because of their non-biodegradability [10]. Pentlavalli et al. reviewed self-assembled peptide nanoparticles for DDS applications [21]. Peptides form well-defined nanostructures, such as nanotubes, nanofibers and so on, through electrostatic interactions, hydrophobic interactions, hydrogen bonding, and π-π stacking, which carry and transport the drug to the affected area. Thus, techniques that produce nanoparticles with precisely controlled particle sizes, surface charge, cell adhesion, and biodegradability are required to accumulate nanoparticles to the tumor and eliminate from the body.

A radiation modification technique to produce various bio-devices from biopolymers has been recently reported [22]. This technique has several advantages: It uses a three-dimensional network structure construction without the need of a toxic chemical crosslinker, it is sterile, and it maintains biocompatibility with no significant molecular structural changes. Biocompatible polydimethylsiloxane (PDMS) is micro-fabricated by ionizing radiation to produce a microfluidic device that could be applied to practical diagnostics [23,24]. Polysaccharides have been modified by ionizing radiation for application as bio-actuators and 3D gel dosimeters [21,25,26] and micro-fabricated to be a culture vessel for application in the biological and medical research fields [27,28]. Moreover, as radiation-crosslinked polysaccharides, hydrogels can be swelled in water and adsorbed in inorganic substances and are used in many applications including cosmetics, wound dressings, scaffold materials for cell culture, and metal adsorbents. On the other hand, radiation modification of proteins has been reported since the 2000s [29,30,31]. The radiation crosslinking mechanism of gelatin, a protein-based material, was investigated in detail by amino acid analysis and fluorescent HPLC in our previous work [32]. Our results revealed that phenylalanine (Phe), tyrosine (Tyr), and histidine (His) residues in the gelatin were radiation crosslinking points, while the other amino acid residues were barely decomposed by irradiation. The reported radiation crosslinking technique modified the gelatin while maintaining the cell adhesion active sequence and retaining high transparency. Radiation crosslinking was further studied for the development of a gelatin scaffold for tissue engineering. Protein nanoparticles within the size range 10–200 nm have been reported in previous studies [33,34]. The authors prepared 6–20 nm nanoparticles by γ-ray irradiation of a gelatin solution and loaded them with Gd to develop an MRI contrast agent that does not penetrate the blood–brain and blood–cerebrospinal fluid barriers and is quickly excreted from the kidney system [35]. Notably, it is important to ensure that the appropriate physical and chemical properties allow for accumulation in the tumor as a probe.

The aim of this study was to form a nanoparticle platform suitable for diagnostic and DDS applications. Peptide nanoparticles with a particle size of ~100 nm, controlled surface potential, no toxic crosslinking agent, and biodegradability were developed by the radiation crosslinking technique. The peptides comprised radiation crosslinking amino acid residues (Phe, Tyr, and His), which formed a crosslinked structure by the radiation-induced bimolecular reaction. Glycine (Gly), which is the major component native protein but not related to protein radiation crosslinking, was also selected as a peptide component. The cell organelles recognized a minimum of three amino acid sequences. Moreover, screening of the ideal synthetic peptide sequences for application to the design of cell culture scaffolds has shown that tetrameric and pentameric peptides are long enough to exhibit good cell adhesion [36]. Therefore, we employed five amino acid residues per peptide and designed and synthesized six peptides to form a radiation-crosslinked nanoparticle platform.

## 2. Materials and Methods

### 2.1. Materials

2-Chlorotrityl chloride resin (reaction point density: 1.6 mmol g^−1^; Watanabe Chemical Industries, Hiroshima, Japan) was used as an insoluble resin carrier. 9-Fluorenylmethyloxycarbonyl-Phe (Fmoc-Phe, >99%, AAPPTEC), 9-fluorenylmethyloxycarbonyl-tyrosine (Fmoc-Tyr, >99%, AAPPTEC), *N*-Fmoc-*N*-trityl-L-histidine (Fmoc-His, >99%, Sigma-Aldrich, St. Louis, MA, USA), and 9-fluorenylmethyloxycarbonyl-glycine (Fmoc-Gly, AAPPTEC) were used as an extension of the peptide chain. 1-[Bis(dimethylamino)methyliumyl]-1H-1,2,3-triazolo [4,5-b]pyridine-3-oxide hexafluorophosphate (HATU, AAPPTEC) and diisopropylethylamine (DIEA, Fujifilm Wako Pure Chemicals, Osaka, Japan) were used as condensing agents. *N*,*N*-dimethylformamide (DMF), dichloromethane (DCM), methanol (MeOH), piperidine, trifluoroacetic acid (TFA), triisopropylsilane (TIS), and diethyl ether (Et_2_O), all purchased from Fujifilm Wako Pure Chemicals, were used for washing, cutting, and dissolving the Fmoc reagent. Ninhydrin and ethanol, also purchased from Fujifilm Wako Pure Chemicals, were used for the ninhydrin color test.

### 2.2. Solid-Phase Synthesis of the Peptides

The six designed and synthesized peptides are listed in Table 1. Dried 2-chlorotrityl chloride resin (100 mg) was set into a solid-phase column (RT5M100, HiPep Laboratories, Kyoto, Japan) coupled with a PTFE two-way stopcock (RTV-SF2, HiPep Laboratories) and subsequently washed with 1 × 10^−3^ dm^3^ DCM for 1 min. Next, Fmoc-amino acid (0.480 mmol) was weighed and dissolved in 1 × 10^−3^ dm^3^ DCM/DMF mixed solvent at a 9:1 volume ratio. The Fmoc-amino acid solution was added to the solid-phase column and stirred with a shaker (ASCM-R 50, ASONE) at 1200 rpm for 50 min. The column was then washed three times each with a DCM:MeOH:DIEA mixture (17:2:1 volume ratio), DCM, and DMF.

Piperidine (20 wt.%) in DMF was added to the column containing the sample and stirred at 1200 rpm for 30 min using a shaker. The column was washed three times with DMF. Subsequently, a color reaction test with ninhydrin ethanol solution (0.7 wt.%) was performed to confirm the removal of the Fmoc group from the sample by piperidine. Ninhydrin reacts with the *N*-terminal amine of the peptide to form Ruhemann’s purple, which is light purple. Next, Fmoc-amino acid in DMF (0.48 mmol), DIEA (0.978 mmol), and HATU (0.489 mmol) were added to the column and stirred with a shaker at 1200 rpm for 120 min. The column was washed three times with DMF, after which the color reaction test was performed to confirm elongation of the Fmoc-amino acids.

The solid-phase column was washed four times with DCM and then dried by aeration overnight. A TFA:Millipore Milli-Q water (total organic carbon: 4 ppb, electric resistance: 1.8 MΩ cm):TIS mixed solution (95:2.5:2.5 volume ratio; 1 × 10^−3^ dm^3^) was next added and the mixture was stirred at 1200 rpm for 30 min. The peptides released from the resin in the solid phase column following the addition of TFA were concentrated approximately fivefold by blowing N_2_ gas for 10 min. Et_2_O was then added to the concentrated peptide solution to produce a precipitate, which was centrifuged for 5 min at 4 °C at a rotation speed of 3000 rpm using a centrifuge (CF15RX, Hitachi, Ltd., Tokyo, Japan). Next, Et_2_O (10 × 10^−3^ dm^3^) was added to the peptide residue and then decanted. After repeating this procedure twice, the peptide residue was vacuum-dried at 30 °C overnight, using a vacuum dryer (Tokyo Glass Instruments) connected to a thermostatic bath and pump.

### 2.3. Preparation and Irradiation of the Synthetic Peptide Aqueous Solution

The synthesized peptides were dissolved to 0.1 wt.% in Millipore Milli-Q water. The sample solutions were irradiated under aerated conditions at 25 °C with γ-ray doses in the range 5–15 kGy (Gy = J kg^−1^) and dose rates (DR) in the range 0.5–10 kGy h^−1^, using a ^60^Co γ-ray source at the Takasaki Advanced Radiation Research Institute, National Institutes for Quantum and Radiological Science and Technology (QST). Dosimetric measurement of the absorbed dose and DR were carried out with an alanine-based dosimeter, according to literature procedures [27].

### 2.4. Kinetics Study

Phe (Tokyo Chemical Industry Co., Ltd., Tokyo, Japan; >98.0%) was used without further purification as the reference material, to evaluate the rate constant of the reaction of the peptides with the hydroxyl (·OH) radicals. The peptides and Phe (0.5 mmol dm^−3^ each) were dissolved in Milli-Q water, and the mixed solution was subjected to γ-ray irradiation at the dose range 1–225 Gy.

### 2.5. HPLC and Ion Chromatography Analysis

The synthesized peptides in water (100 × 10^−6^ dm^3^) before and after irradiation were purified using a 0.22 µm membrane filter (Millipore) and then analyzed using a high-performance liquid chromatograph connected to a fluorescence detector (2475, Waters) and a mass spectrometer (Shimadzu, LCMS-2020). Next, 0.1 wt.% formic acid and acetonitrile were eluted into the column (Shodex ODS-DE613) with a gradient mixture ratio (formic acid/acetonitrile = 95/5 to 5/95) and a flow rate of 1.0 × 10^−3^ dm^3^ min^−1^ for HPLC-MS analysis.

TFA-containing peptide samples were analyzed with an ion chromatography system (Shimadzu, SCL-10A) using an ion-exchange column (Shodex IC SI-90 4E) at 40 °C. A mixed solution of sodium carbonate (1.8 mmol dm^−3^) and sodium hydrogen carbonate (1.7 mmol dm^−3^) was used as the eluent with a flow rate of 1 × 10^−3^ dm^3^ min^−1^.

### 2.6. Dynamic Light Scattering (DLS) and ELS Measurement

The particle size and zeta potential of the peptide solution in water and phosphoric acid buffer solution before and after γ-ray irradiation were respectively measured by DLS and ELS (Zetasizer, Malvern PANalytical, Worcestershire, UK) at room temperature (25 ± 1 °C). The light scattering intensity of the peptide nanoparticles for the DLS measurement was evaluated with a standard polystyrene (PS) solution (1 × 10^9^ particles at 800 nm; 3K800; Thermo Fisher Scientific Inc., Waltham, MA, USA). A mixture of the irradiated peptide solution and internal PS standard solution was prepared in a volume ratio of 99:1. Furthermore, the nanoparticle stabilities in water and phosphoric buffer solution (PBS; pH = 7.4; Gibco^TM^) were observed at 37 °C for six days.

### 2.7. Biodegradability

The biodegradability of the peptide nanoparticles in a buffer solution containing enzymes was next elucidated. The peptide nanoparticle solution (0.01 dm^3^) was added to 0.01 dm^3^ of an aqueous solution of 0.02 mol dm^−3^ sodium hydrogen carbonate (guaranteed reagent grade, Fujifilm Wako Pure Chemical), 0.001 mol dm^−3^ calcium chloride (90%, Fujifilm Wako Pure Chemical), and 0.01 wt.% protease (from Aspergillus oryzae, Tokyo Chemical Industry, Tokyo, Japan). The mixed solutions were incubated at 37 °C for six days, after which the absorbances of the treated solutions were measured with a spectrophotometer (U-3310 spectrophotometer, Hitachi). The degradation rate was determined from the absorbances at 273 nm using Equation (1).

Degradation rate = Abs1/Abs0 × 100(1)

where Abs1 and Abs0 are the absorbances of the filtrate and filtrate, respectively.

### 2.8. Cellular Uptake Test

Fluorescent staining of the peptide nanoparticle solution was carried out using a HiLyte Fluor^TM^ 555 Labeling Kit-NH_2_ (LK14, Dojindo Laboratories, Kumamoto, Japan). The labeled peptide solution was poured into the ultrafiltration system (100 kDa; Millipore) and washed threefold with PBS under centrifugation (CF15RN, HIMAC) conditions. Fluorescent spectra of the labeled peptide nanoparticle solutions were then obtained with a fluorescent spectrophotometer (Shimadzu, RF-5300).

HeLa human cervical carcinoma epithelial cells (RCB0007, Bioresource Center, RIKEN, Ibaraki, Japan) were cultured in minimum essential Eagle medium (M5650, Sigma-Aldrich) with 10 vol % fetal bovine serum (SH30910.03, GE Healthcare Japan, Tokyo, Japan), 100 × 10^3^ U dm^−3^ penicillin, 100 mg dm^−3^ streptomycin, and 2 mmol dm^−3^ L-glutamine (10378016, Thermo Fisher Scientific). HeLa cells (4 × 10^4^) were seeded on the glass-based dish (3911-035, AGC Techno Glass, Shizuoka, Japan) and incubated at 37 °C in 5% carbon dioxide for two days. After removing the medium by suction, Opti-MEM cell culture medium (31985070, Thermo Fisher Scientific; 75 × 10^−6^ dm^3^) was mixed with fluorescent-labeled peptide nanoparticles in PBS (75 × 10^−6^ dm^3^) and then placed into the dish of HeLa cells. After incubation at 37 °C in 5% carbon dioxide for 2 h, the medium was changed to Opti-MEM medium (150 × 10^−6^ dm^3^) containing 1 mg dm^−3^ Hoechst 33,342 (H342, Dojindo Laboratories) and 5 mg dm^−3^ CellMask Green plasma membrane stain (C37608, Thermo Fisher Scientific) and incubated at 37 °C in 5% carbon dioxide for 5 min. After washing with 1 × 10^−3^ dm^3^ PBS twice, 4 wt.% paraformaldehyde in PBS (163-20145, Fujifilm Wako Pure Chemicals) was added to the HeLa cells in the dish, which were then incubated at room temperature for 10 min. The fixed cells were washed twice with 1 × 10^−3^ dm^3^ PBS and incubated in 2 × 10^−3^ dm^3^ PBS for subsequent microscopic observation.

The glass-based dish was placed on the stage of an upright microscope (BX51WI, Olympus, Tokyo, Japan) with a confocal disk scan unit (DSU, Olympus), and observed using a X60 water immersion lens (LUMPLFLN60XW, Olympus) and a COMS camera (ORCA-Flash4.0 V3, Hamamatsu Photonics, Shizuoka, Japan). Nuclei labeled with Hoechst 33,342 were excited at 382–393 nm and observed at 417–477 nm, while cell membranes labeled with CellMask Green were excited at 460–480 nm and observed at 495–540 nm. Moreover, the nanoparticles labeled with fluorescent reagent were excited at 535–555 nm and observed at 570–625 nm.

## 3. Results and Discussion

### 3.1. Peptide Synthesis

Six peptides were designed based on the radiation crosslinking mechanism of gelatin [32] (Table 1) and synthesized by solid-phase extraction methods to obtain white powders. In these peptides, Phe, Tyr, and His are radiation crosslinking sites, while Gly is not involved in crosslinking. The six peptide powders were each dissolved in Milli-Q water to prepare 0.1 wt.% peptide aqueous solutions. The mass spectra of these peptide solutions displayed a single peak of the parent peptide molecule. Moreover, ion chromatography revealed residual TFA in these peptides in the range 1–4 wt.% and the nanoparticles were separated using ultrafiltration after γ-ray irradiation.

### 3.2. Kinetics of the Synthesized Peptides in Water by γ-ray Irradiation

The mechanism for radiation crosslinking of amino acids and peptides in water has been the subject of qualitative analyses in previous studies. When the peptides in solid-state were irradiated with γ-rays, negligible crosslinkage occurred, indicating that the direct effect of γ-ray irradiation on the crosslinking of peptides and gelatin is small [26]. In the case of aqueous solutions of proteins, radiation crosslinking was attributed to the reaction of aromatic amino acids with the radiation-induced reactive species in water [29,30,31,32,35]. The reactive species produced by the radiolysis of water are ·OH radicals, hydrated electrons (e^−^_aq_), and hydrogen atoms (Equation (2)).

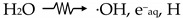
(2)

The main chain of the protein in water is oxidized by ·OH radicals to produce protein radicals [37]:

RCONHCHR_2_ (protein) + ·OH → H_2_O + RCONH ·CR_2_ (3)

Under oxygenated conditions, these protein radicals react with dissolved oxygen to produce peroxyl radicals, which subtract hydrogen atoms as follows:

RCONH·CR_2_ + O_2_ → H_2_O + RCONH C(OO·)R_2_ (4)

RCONH C(OO·)R_2_ + ·H → RCONH C(OOH)R_2_ (5)

The formed peroxyl peptides in water then induce oxidative main-chain degradation as illustrated in Equation (6):

RCONH C(OOH)R_2_ + 2H_2_O → RCOOH + NH_3_ + R_2_CO + H_2_O_2_ (6)

Most e^−^_aq_ react with dissolved oxygen to produce O_2_^−^ ions, which display lower reactivity than ·OH radicals. On the other hand, the aromatic rings in the protein aromatic amino acid residues are oxidized by the ·OH radicals from water radiolysis to produce stable protein radicals (Equation (7)), which result in crosslinking of the protein via bimolecular reactions (Equation (8)) [32,37,38].

C_6_H_5_-CH_2_CH(NH_2_)COOH + OH → ·C_6_H_4-_CH_2_CH(NH_2_)COOH + H_2_O (7)

2 · C_6_H_4_-CH_2_CH(NH_2_)COOH →                            HOOCCH(NH_2_)CH_2_-C_6_H_4_-C_6_H_4_-CH_2_CH(NH_2_)COOH (8)

Therefore, radiation crosslinking of a protein in water is primarily induced by the ·OH radicals produced from water radiolysis.

In this study, the reactivity of the synthesized peptides with ·OH radicals was evaluated by the competition reaction method [39]. Phe can be regarded as a standard to estimate the relative rate constant of the synthesized peptides with ·OH radicals:

Peptide + ·OH → stable products(9)

Phe + ·OH → stable products(10)

where *k*_Peptide_ and *k*_Phe_ are the reaction rate constants of the ·OH radicals with the peptide and Phe, respectively. At constant DR, the decomposition yield of the peptide can be expressed by the rate constant and concentration of the ·OH radicals as

(11) $$-\frac{d[\mathrm{Peptide}]}{dD}\;=\;-\;\frac{1}{\;DR}\frac{d[\mathrm{Peptide}]}{dt}\;=\;-\;\frac{1}{DR}k_{\mathrm{Phe}}\left[\mathrm{Peptide}][\cdot \mathrm{OH}\right]$$

where *D* and *t* are the dose and irradiation time, respectively. When the initial concentrations of both solutes are the same, the peptide-to-Phe decomposition ratio is the ratio of the rate constants under the same dose rate irradiation:

(12) $$-\frac{d\;[\mathrm{Peptide}]}{d\;D}/-\frac{d\;[\mathrm{Phe}]}{d\;D}{\;=\;k}_{\mathrm{Peptide}}{/\;k}_{\mathrm{Phe}}$$

**Figure 1:**
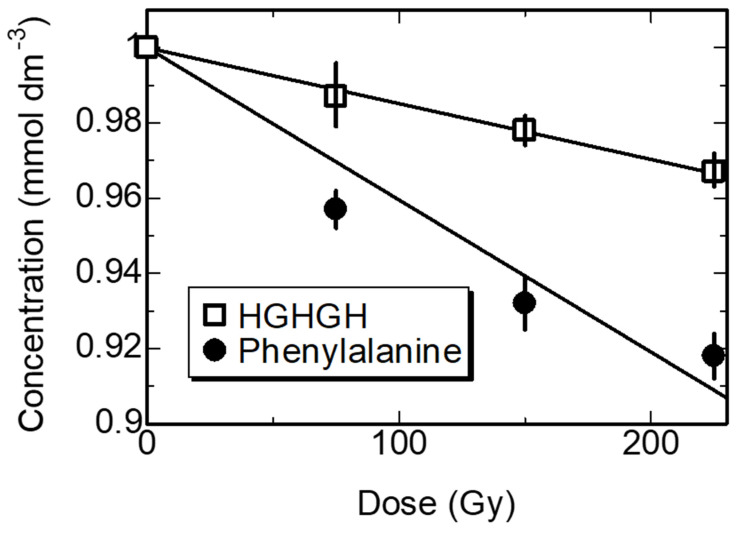
Decomposition of HGHGH and phenylalanine (Phe) in water by γ-ray irradiation at room temperature.

Mixed solutions of the peptide with Phe (1 × 10^−3^ mol dm^−3^ each) were irradiated at a lower γ−ray dose region to eliminate the effect of the secondary reactions as much as possible. Figure 1 shows the decrease in the HGHGH and Phe concentrations with increasing absorbed dose. The ratio of the decomposition yield of HGHGH to that of Phe was determined from the slope of the fitted lines of the initial decomposition curves. The rate constant of HGHGH with ·OH radicals was estimated at 2.6 × 10^9^ mol^−1^ dm^3^ s^−1^, and those of the other peptides with ·OH radicals were estimated by the same method (Table 2).

The rate constants of these aromatic peptides with ·OH radicals were similar to those of the amino acids (Table 2). Although the synthesized peptides comprised three aromatic groups per molecule, they did not display a higher reactivity than those of the corresponding amino acids. This was because the reactivity of the peptides with ·OH radicals was suppressed by steric hindrance induced by the Gly sandwiched between the aromatic amino acid residues. Nevertheless, the rate constants of the peptides with ·OH radicals were ~10^9^ mol^−1^ dm^3^ s^−1^, and the radiation crosslinking of the peptides induced by the bimolecular reaction of aromatic amino acids residue (Equation (8)) was assumed to be carried out effectively. The rate constant of Gly with ·OH radicals is reported at 1.7 × 10^7^ mol^−1^ dm^3^ s^−1^ [40]. There are no high-electron-density substituents for Gly, and the contribution to radiation crosslinking is lower than that of the other aromatic amino acid residues.

### 3.3. Production of Peptide Nanoparticles by Ionizing Radiation

In the mass spectrometric analysis of the aqueous solutions of the peptides containing one to three Phe units after 2.5 kGy irradiation, only the monomer peak (m/z = 394) was detected for peptide FG4, and no multimerization reaction was observed. For the peptide FG3F, both the monomer (m/z = 484) and dimer (m/z = 967) peaks were observed. In addition, monomer (m/z = 573), dimer (m/z = 1147) and trimer (m/z = 1721) peaks were observed for the peptide FGFGF. This suggests that peptides containing one or two amino acid residues that are thought to contribute to the crosslinking reaction are unlikely to undergo multimerization. Therefore, the peptides containing three residues, namely Phe, Tyr, and His, were found to be more suitable for the production of nanoparticles and used in the subsequent experiments.

**Figure 2:**
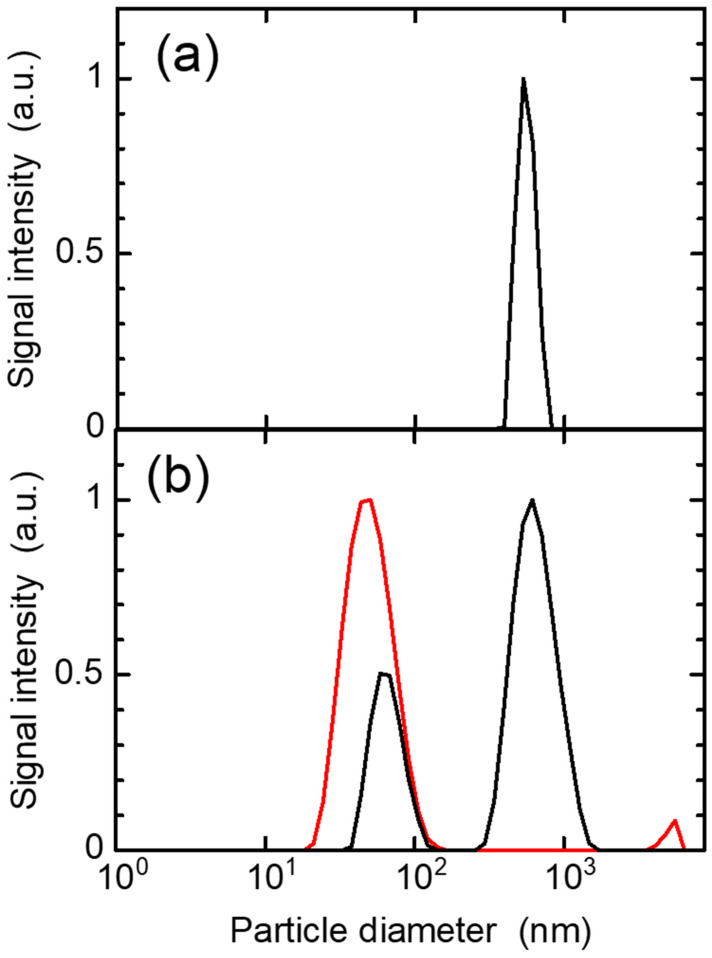
Light scattered intensity of 0.1 wt.% HGHGH aqueous solution (**a**) unirradiated and (**b**) irradiated with 10 kGy γ-rays with (black) and without (red) internal standard.

When 0.1 wt.% aqueous solutions were prepared with the peptides FGFGF, YGYGY, and HGHGH and irradiated with 5 kGy γ-rays, white turbidity was observed. Thus, when the unirradiated and irradiated samples of the aqueous peptide solution were exposed to laser light, the irradiated sample showed light scattering, owing to the cloudy component. The particle size was measured by DLS. Figure 2a shows the particle size distribution of the unirradiated aqueous HGHGH solution with the PS standard. The PS signal was observed at 800 nm; however, no other peak originating from HGHGH was observed. Similarly, no peak was observed for the peptides FGFGF and YGYGY.

**Scheme 1:**
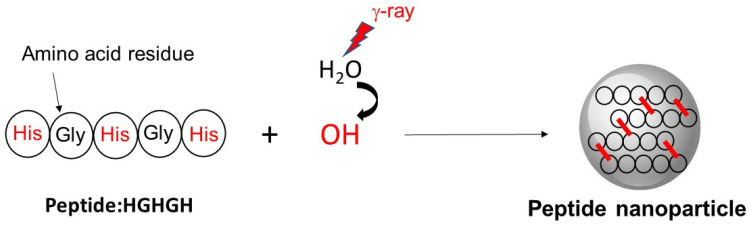
Production of peptide nanoparticle from HGHGH in water by γ-ray irradiation.

Next, the light scattering spectra of the FGFGF, YGYGY, and HGHGH aqueous solutions irradiated with 10 kGy were recorded on DLS. A single peak at ~50 nm was observed for the 10 kGy irradiated HGHGH aqueous solution, while two peaks were observed for the irradiated solution with the internal standard (Figure 2b). The peak observed near 800 nm was assigned to the internal standard, while the peak at 50 nm shifted to a larger particle size following the addition of the internal standard. The chemical bonding between the peptide nanoparticles and PS particles is unlikely, and thus the peak positions of each component are apparently shifted slightly from the original ones by two-component analysis. Therefore, HGHGH nanoparticles at 50 nm were detected from an HGHGH aqueous solution after γ-ray irradiation as shown in Scheme 1. Nanoparticles were also observed for the peptides FGFGF and YGYGY. The values of polydispersity index (PDI) of these three type peptide nanoparticles were 0.2 to 0.4 for DLS measurement.

**Figure 3:**
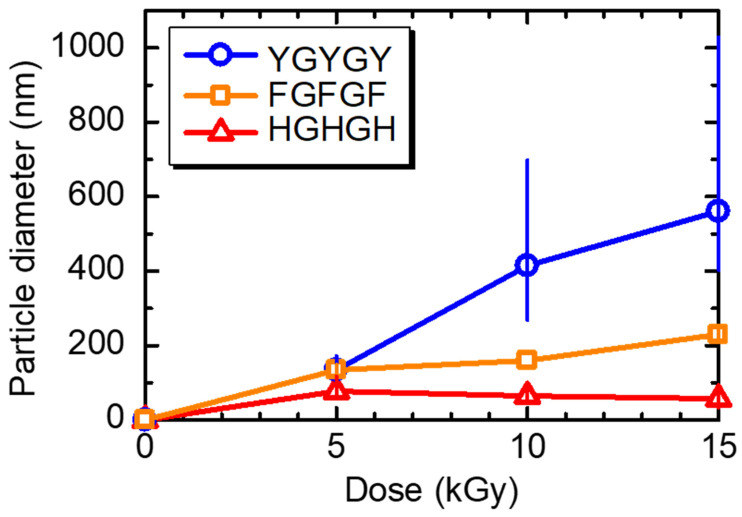
Dose dependence of the nanoparticle size obtained from three peptides by γ-ray irradiation.

The dose dependence of the nanoparticle size of the three peptides in γ-ray irradiation was next investigated (Figure 3). The particle diameters of YGYGY and FGFGF increased with the increasing absorbed dose, while that of HGHGH was almost constant or decreased at doses ˃5 kGy. The rate constants of these peptides with ·OH radicals were higher in the order FGFGF ˃ YGYGY ˃ HGHGH (Table 2); however, their particle size was higher in the order YGYGY ˃ FGFGF ˃ HGHGH. The formation of the peptide particle is considered to be affected not only by the rate constant but also the aggregation based on their hydrophobicity. The particle size range of YGYGY was controlled in the range 130–550 nm by the absorbed dose, while that of FGFGF was 140–220 nm. As mentioned above, the polydispersity of each particle is low. However, the variation of particle size increased with higher dose, i.e., longer irradiation time. It is considered to be due to aggregation caused by the lower surface charge and hydrophobic interactions. In this case the bimolecular reaction of YGYGY was considered to occur more effectively than that of FGFGF, based on the reports that the *G*-value for the formation of dihydroxybiphenyl in an aqueous phenol solution was 0.24 μmol dm^−3^ Gy^−1^ [41]. This value was twice as high as that for biphenyl formation from an aqueous benzene solution (0.12 μmol dm^−3^ Gy^−1^) [42].

The dose dependence of the particle size appears to be different at values above and below 5 kGy. Moreover, the particle sizes of the peptides are considered to be affected by the dissolved oxygen concentration based on the bimolecular reaction of the peptide (Equation (8)). Thus, the addition of ·OH radicals to Phe and Tyr was enhanced by the dissolved oxygen [32], while their bimolecular reaction (Equation (8)) was suppressed. The concentration of dissolved oxygen is ~250 μmol dm^−3^ under aerated conditions, while the radiation chemical yield (G-value) of the ·OH radicals from water radiolysis is 280 μmol dm^−3^ kGy^−1^. Thus, the bimolecular reaction is more advantageous than ·OH addition and degradation of the peptide (Equation (4)) above 1 kGy when the dissolved oxygen is completely reacted with the peptide and consumed.

### 3.4. Stability and Biodegradability of the Peptide Nanoparticles

**Figure 4:**
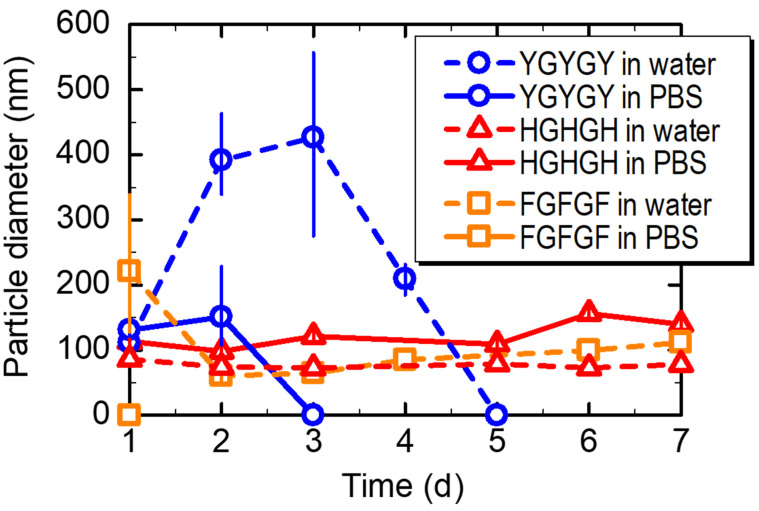
Stability of the YGYGY and HGHGH nanoparticles in water and in phosphoric buffer solution (PBS) simulating in vivo conditions.

The particle size stabilities of the nanoparticles were investigated by DLS in water and in PBS to simulate in vivo conditions (Figure 4). The FGFGF nanoparticles in both water and PBS broke down after one day. The YGYGY nanoparticles in PBS lasted for two days before they began to degrade and no particles were observed on the third day. The YGYGY particles in water were observed for a few days; however, decomposition and aggregation eventually occurred and no particles were observed on the fifth day. Both the FGFGF and YGYGY nanoparticles were considered to be decomposed by the dissolved oxygen because Phe and Tyr residues are easily oxidized by dissolved oxygen. On the other hand, the HGHGH nanoparticles were stable in water and PBS for six days because the His residue is difficult to oxidize. Moreover, the measured surface potential of the HGHGH nanoparticles was +23.4 mV (Table 3), revealing that the electrostatic repulsion between these nanoparticles hindered their aggregation.

**Figure 5:**
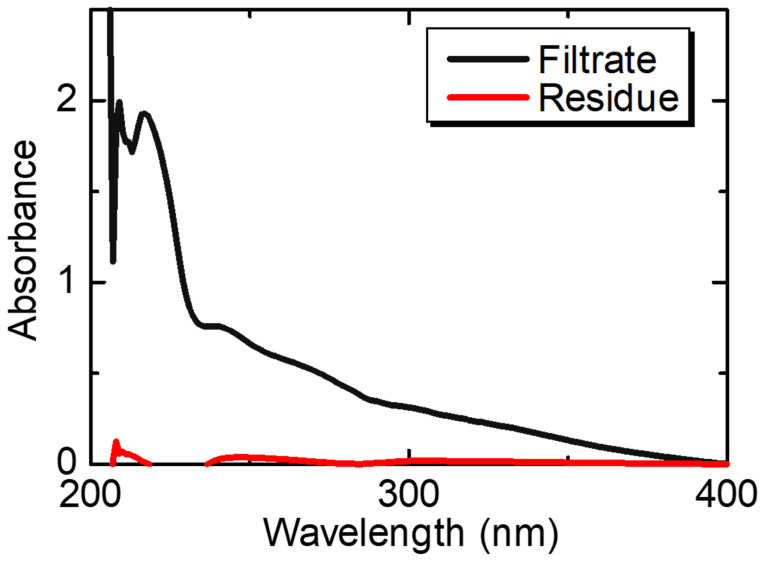
Absorbance of the HGHGH nanoparticle filtrate and residue after protease treatment for six days.

It is important to evaluate the biodegradability in vivo to promote medical applications such as safe diagnostic methods. Thus, HGHGH nanoparticles of smaller particle size and higher stability than those of FGFGF and YGYGY nanoparticles were next degraded with human protease to evaluate their biodegradability. The absorbance of the residue containing HGHGH nanoparticles and filtrate containing biodegradation products was then measured after ultrafiltration (Figure 5), and the background absorbance (protease and PBS buffer) was subtracted from the measure values.

The volume rate of the residue/filtrate was 0.2/2.8 mL/mL, and the calculated degrees of residue and filtrate concentration were 15 and 1.1, respectively. The absorbance values of the residue and filtrate at 273 nm were 0.49 and 0.01, respectively, and were assigned to the HGHGH aromatic group. These results indicated that protease degrades the peptide bonds of these HGHGH nanoparticles by 98% within six days. The radiation-crosslinked HGHGH nanoparticles were biodegraded effectively because their side chains are crosslinked and the original chemical structure of their main chain is preserved. Because the His dimer produced after biodegradation is smaller than the glomerular meshwork (7 nm) of the kidney [15,20], it easily passes through this network and can be rapidly excreted from the blood. We therefore concluded that HGHGH nanoparticles do not accumulate in the blood. Indeed, they are biodegraded by the enzymes within a week, after which they are rapidly excreted from the body. The biodegradability of HGHGH is similar to that of the radiation-induced nanoparticles from gelatin, which displayed 95% degradation in seven days by protease [35]. The stability and biodegradability of HGHGH nanoparticles are not expected to be affected by staining.

### 3.5. Cellular Uptake Test for the Fluorescent-Labeled Peptide Nanoparticles

**Figure 6:**
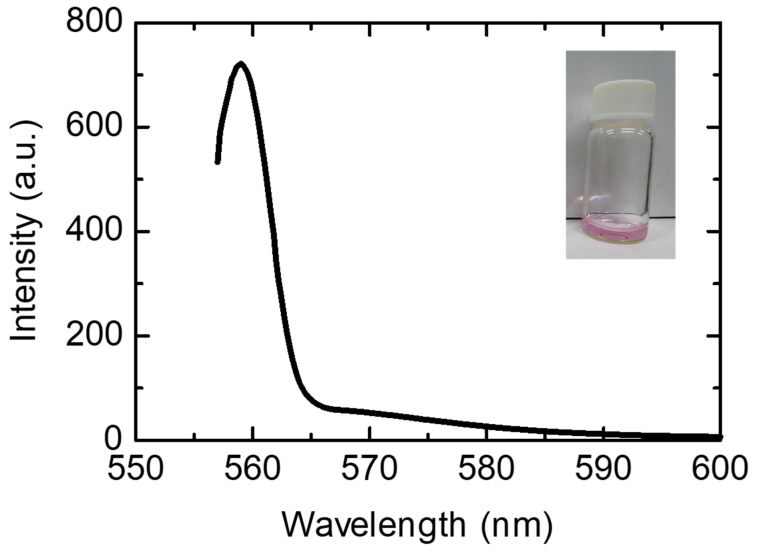
Aqueous solution containing fluorescent stained HGHGH nanoparticles (inset photo) and its fluorescence spectrum (main figure).

HGHGH nanoparticles were successfully labeled with fluorescent reagent as shown in Figure 6. The fluorescent labeling reagent comprised an *N*-hydroxysuccinimide (NHS) group, which rapidly reacted with a peptide amino group. Thus, the HGHGH surface potential changed from +23 mV to −14 mV after fluorescent labeling (Table 3). The surface potential did not change when double the amount of fluorescent reagent was used, indicating that most of the HGHGH amino acid groups were labeled by the initial amount of fluorescent reagent.

**Figure 7:**
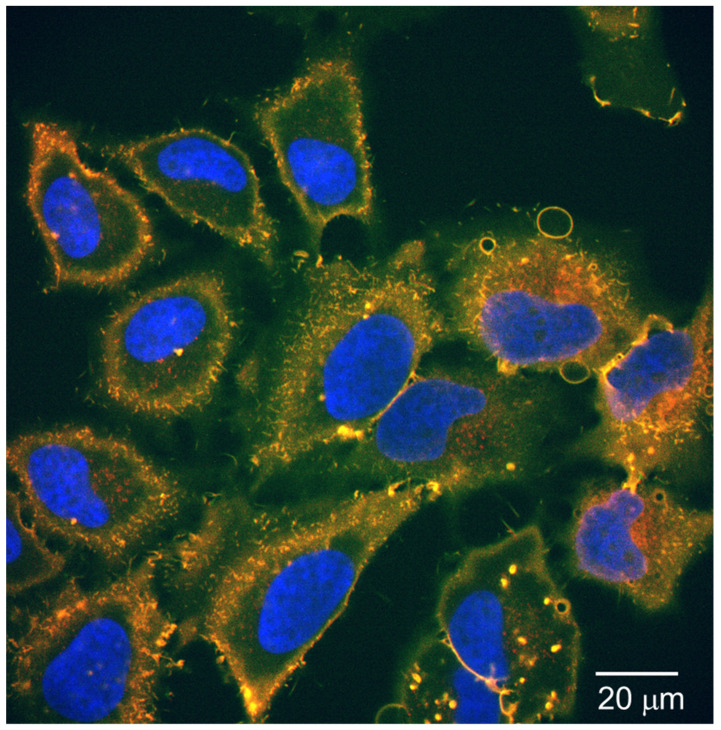
Confocal image of red-fluorescent 80 nm HGHGH nanoparticles in HeLa cells. Blue: nuclei, yellow: cell membranes, and red: nanoparticles.

The cellular uptake test of the fluorescent-labeled HGHGH nanoparticles was carried out using HeLa cells (Figure 7). The confocal images revealed that the HGHGH nanoparticles were distributed inside the cells. The negative surface of the fluorescent-labeled HGHGH nanoparticles allows them to attach to the surface of the HeLa cells, whereby they are uptaken into the cell via endocytosis [18]. In our previous study, we revealed that the radiation crosslinking technique does not consume the amino and carboxyl groups of the peptides [32]. Moreover, since there are many fluorescent dyes that can be attached to the amino groups, the fluorescent dye can be changed depending on the cell observation conditions. It is also possible to produce HGHGH nanoparticles with enhanced positive charge by binding a fluorescent-labeled reagent to their carboxyl groups. Furthermore, a chelator can be attached to the HGHGH amino or carboxyl group and applied to various agents including Gd-based MRI contrast, boron neutron capture therapy (BNCT), and RI-based PET agents.

## 4. Conclusions

Peptides composed of Phe, Tyr, His, and Gly were designed and synthesized based on the radiation crosslinking mechanism of proteins. Thus, peptide nanoparticles, with a controlled size range (60–550 nm), which displayed biodegradability and a surface potential of +23 mV, were produced by the radiation crosslinking technique in the absence of toxic crosslinking agents. The HGHGH peptide nanoparticles, with a diameter of 80 nm, were stable in water and PBS for six days because of the electrostatic repulsion from the surface positive charge. The surface charge of the peptide nanoparticles could be changed to −14 mV by fluorescent staining, thereby allowing them to invade HeLa cells. The radiation crosslinking technique can be employed to create a nanoparticle platform with great potential for application as nanosensors and nano-DDS carriers for tumor diagnosis and therapy.

## Figures and Tables

**Table 1:** Composition of the designed peptides.

Abbreviation	Peptide Sequence
HGHGH	His-Gly-His-Gly-His
YGYGY	Tyr-Gly-Tyr-Gly-Tyr
FGFGF	Phe-Gly-Phe-Gly-Phe
FG4	Phe-Gly-Gly-Gly-Gly
FG3F	Phe-Gly-Gly-Gly-Phe
G5	Gly-Gly-Gly-Gly-Gly

His: histidine, Gly: glycine, Tyr: tyrosine, Phe: phenylalanine.

**Table 2:** Rate constant of the synthesized peptides with ·OH radicals in water. The rate constants of the amino acids with ·OH radicals have been referred to from Masuda et al., 1973 [40].

Amino Acids and Peptides	Abbreviation	Rate Constant with ·OH Radicals (mol^−1^ dm^3^ s^−1^)
Phenylalanine	F	7.2 × 10^9^
Histidine	H	4.3 × 10^9^
Tyrosine	Y	10.5 × 10^9^
Glycine	G	1.7 × 10^7^
His-Gly-His-Gly-His	HGHGH	2.6 × 10^9^
Tyr-Gly-Tyr-Gly-Tyr	YGYGY	4.1 × 10^9^
Phe-Gly-Phe-Gly-Phe	FGFGF	6.8 × 10^9^

**Table 3:** Zeta potential of the produced peptide nanoparticles before and after fluorescent staining.

Peptides	Before Fluorescent Staining /mV	After Fluorescent Staining /mV
HGHGH	+23.4	−13.9
HGHGH (double amount)	+23.4	−14.9
